# Evidence for the Use of Multiple Mechanisms by Herpes Simplex Virus-1 R7020 to Inhibit Intimal Hyperplasia

**DOI:** 10.1371/journal.pone.0130264

**Published:** 2015-07-01

**Authors:** Susan McCormick, Qi He, Jordan Stern, Nikolai Khodarev, Ralph Weichselbaum, Christopher L. Skelly

**Affiliations:** 1 Section of Vascular Surgery, Department of Surgery, University of Chicago, Chicago, Illinois, United States of America; 2 Department of Radiation and Cellular Oncology and Ludwig Center for Metastasis Research, University of Chicago, Chicago, Illinois, United States of America; University of Illinois at Chicago, UNITED STATES

## Abstract

Intimal hyperplasia (IH) is the primary cause of vein bypass graft failure. The smooth muscle cell (SMC) is a key element of IH as it phenotypically switches from a contractile to a synthetic state which can become pathological. R7020, which is an engineered strain of Herpes Simplex Virus-1, inhibits IH in animal models. Although it has many characteristics which make it a strong candidate for use as a prophylactic agent how it inhibits IH is not well understood. The objective of this study was to identify modes of action used by R7020 to function in blood vessels that may also contribute to its inhibition of IH. The cytopathic effect of R7020 on SMCs was determined *in vitro* and in a rabbit IH model. *In vitro* assays with R7020 infected SMCs were used to quantify the effect of dose on the release kinetics of the virus as well as the effects of R7020 on cell viability and the adhesion of peripheral blood mononuclear cells (PBMCs) to SMCs in the absence and presence of tumor necrosis factor alpha (TNF-α). The observed cytopathic effect, which included R7020 positive filopodia that extend from cell to cell and the formation of syncytia, suggests that R7020 remains cell associated after egress and spreads cell to cell instead of by diffusion through the extracellular fluid. This would allow the virus to rapidly infect vascular cells while evading the immune system. The directionality of the filopodia *in vivo* suggests that the virus preferentially travels from the media towards the intima targeting SMCs that would lead to IH. The formation of syncytia would inhibit SMC proliferation as incorporated cells are not able to multiply. It was also observed that R7020 induced the fusion of PBMCs with syncytia suggesting the virus may limit the effect of macrophages on IH. Furthermore, R7020 inhibited the proliferative effect of TNF-α, an inflammatory cytokine associated with increased IH. Thus, the results of this study suggest that R7020 inhibits IH through multiple mechanisms.

## Introduction

Approximately 82.6 million Americans have cardiovascular disease. This includes nearly eight million Americans with peripheral artery disease and 16.3 million Americans with coronary artery disease [1]. Angioplasty, stents and bypass grafts are not ideal solutions for these diseases as they can induce pathological vascular remodeling which results in intimal hyperplasia (IH) and the reoccurrence of symptoms. Two prospective randomized phase III clinical trials have demonstrated that short term (1 month–2 years) vein graft failures are the result of intimal hyperplastic lesions [2–8]. Intimal hyperplasia [9] is a pathological process that occurs locally in blood vessels. It is also a multifaceted disease which includes vascular smooth muscle cell (SMC) proliferation and migration, as well as extracellular matrix production and inflammation [10–15]. The SMC plays a critical role in the development of IH. The phenotypic plasticity of this cell, allows it to switch between contractile and synthetic phenotypes in response to changes in the local environment. During the formation of IH SMCs change from a contractile to a synthetic state allowing them to proliferate and synthesize extracellular matrix which are key events in IH formation [16].

Currently, no clinically available therapy targets IH in vein grafts. In light of the failure of traditional vector based delivery strategies, our laboratory has been interested in engineered strains of Herpes Simplex Virus-1 (HSV-1) as prophylactic agents against pathological IH. Of particular interest is the replication competent R7020, a γ_1_34.5-deficient HSV-1 strain. Intimal hyperplasia, vein bypass graft surgical procedures and R7020 have specific characteristics that make this strain particularly well suited for the treatment of vein graft IH. During the surgery the bypass vein is harvested from the patient. At this point the virus can be delivered locally by injecting it into the lumen eliminating the need for systemic administration. Unlike vectors, which require pathologically high pressures to effectively penetrate the vessel wall, R7020 is delivered efficiently under physiological pressures [17–19]. Once in the wall, R7020 preferentially infects vascular SMCs and inhibits cellular proliferation, and will do so in immune-competent hosts [17, 20]. The inhibition of IH by R7020 has been shown to be sustainable in experimental models of vein graft and angioplasty failure [18, 21]. In addition, R7020 treated vessels will re-endothelialize which is critical for vein graft health and function [18]. The side effects of R7020 in humans have also been studied. Several phase I and II human cancer clinical trials, including ones where the virus was arterially injected [22, 23], have shown it to have an excellent safety profile [24, 25]. However, how R7020 inhibits IH is not well established. We have shown that its anti-proliferative effect is via a caspase 3 and mitogen-activated protein kinase kinase (MEK) dependent mechanism [18, 19]. However, IH is multifaceted and R7020 infects and regulates the cellular pathways and functions of a wide range of cell types. Therefore, we hypothesize that R7020 inhibits IH by altering multiple pathways and functions in multiple cell types. The results of this study suggest that R7020 inhibits IH using a wide range of mechanisms.

## Materials and Methods

### Culture of SMCs

Human aortic smooth muscle cells (American Type Culture Collection, Cat. No. ATCC PCS-100-012, Manassas, VA) were cultured and proliferated in Vascular Cell Basal Medium (ATCC PCS-100-030) supplemented with the components of the Vascular Smooth Muscle Cell Growth Kit (ATCC PCS-100-042. Experiments were performed with SMCs at the fifth passage.

### R7020 infection and imaging of SMCs

SMCs were seeded at a density of 1.25 x 10^4^ cell/cm^2^ on glass coverslips (Corning Inc., Cat. No. 2850–18, Corning, NY) and on two glass slides (Fisher Scientific, Cat. No. 12-550B, Waltham, MA) coated with rat tail collagen (BD Cat. No. 3554236 Medford, MA; 10 μg/cm^2^). They were cultured in DMEM medium supplemented with 10% FBS, penicillin (100 units/ml) and streptomycin (100 μg/ml) for three days with media changed every 24 h. On the third day the average cell density on the glass slides was determined by harvesting the SMCs from the two slides with trypsin and performing a cell count using a hemocytometer. This value was used to approximate the cell density on the glass coverslips for determination of the virus multiplicity of infection (MOI) concentration. SMCs were infected with R7020 (a gift from Dr. Bernard Roizman at the University of Chicago) at 1 MOI for 10 min in DMEM supplemented medium with and without tumor necrosis factor-alpha (TNF-α) (10 ng/ml). The cells were then washed twice with PBS and cultured for 24 h in DMEM supplemented medium with and without TNF-α (10 ng/ml), matching infection conditions. After which they were fixed in 4% paraformaldehyde (PFA) in phosphate buffered saline (PBS) for 15 min at room temperature (RT), washed with PBS and permeabilized for 40 min at RT in PBS with 10% human serum, 1% bovine serum albumin and 0.1% Triton X-100. The cells were incubated with a rabbit antibody to HSV-1 infected cell protein 22 (IPC22) (gift from Dr. Bernard Roizman) and a mouse antibody to ICAM-1 (Santa Cruz Biotechnology, Inc., Cat. No. SC-107, Santa Cruz, CA) or with a mouse antibody to HSV-1 US11 (gift from Dr. Bernard Roizman) at 4°C overnight, washed three times for 5 min in TBS with 0.05% TWEEN 20, incubated with a goat anti-rabbit IgG antibody (Life Technologies, Cat. No. A21071, Carlsbad, CA) and a goat anti-mouse IgG antibody (Life Technologies, Cat. No.A11020, Carlsbad, CA) for 1 h at RT, washed three times for 5 min in TBS with 0.05% TWEEN 20 and then mounted on a glass slide using VECTASHIELD mounting medium with DAPI (Vector Laboratories, Cat. No. H-1200, Burlingame, CA). Images were captured with a Leica TCS SP5 II STED laser scanning confocal microscope (Leica Microsystems, Inc., Buffalo Grove, IL) using a 63x objective.

### Animal model

A low flow common carotid artery (CCA) balloon injury IH model was used to investigate the response of cells in injured arteries to R7020. This animal model has been characterized in both rats [12] and rabbits [26]. In this study male New Zealand White Rabbits (3 kg) were utilized as previously described [13, 18]. This study was carried out in strict accordance with the recommendations in the Guide for the Care and Use of Laboratory Animals of the National Institute of Health. The protocol was approved by the Institutional Animal Care and Use Committee at the University of Chicago (protocol number 71179). All surgeries were performed under sodium pentobarbital anesthesia and all efforts were made to minimize suffering. Prior to the end of surgery, the rabbits received one dose of Buprenorphine (10–50mcg/kg) intramuscularly. Thereafter, if there was evidence of pain additional Buprenorphine (5–10 mcg/kg) was administered subcutaneously no less than every 12 h during the 24 h or 3 day experimental time period. The animals were checked twice daily by investigative or clinical staff for pain. They were allowed to recover without reversal of the anesthetic agents. The rabbits were evaluated for their urine and fecal output, food intake and water drinking behavior. At the conclusions of the experiments, the animals were re-anesthetized, the arterial segments were collected and the animals were euthanized using 120 mg/kg IV pentobarbital. There were no incidental animal deaths during this study.

### Carotid artery treatment *in vivo*

For all three experimental conditions, balloon injury low flow (BILF) with R7020 treatment, BILF with no viral treatment and sham treatment, an incision was made and the CCA was dissected away from the surrounding tissue. For the BILF R7020 treatment group the carotid artery was exposed to R7020 at an infection level of 1 x 10^9^ plaque forming units per artery after balloon injury. The virus was suspended in PBS and administered via direct intra-arterial delivery. For the next 10 min the arterial pressure was maintained at the rabbit’s pre-anesthetization systolic pressure. The vessel was then flushed with PBS and blood flow was resumed at a reduced flow rate. For the BILF no viral treatment group, the above procedure was followed except the injected PBS contained no virus. For the sham group, the incision was sutured after dissection of the artery with no further intervention and no alteration of the blood flow rate. Twenty-four hours or three days after the intial surgery the animals were anesthesized. Common carotid arterial segments 4 cm in length were harvested and snap frozen in liquid nitrogen. The BILF R7020 treated group and the sham group consisted of four rabbits each. There were five rabbits in the BILF no viral treatment group.

### Histology and cell counts

Vessel samples were prepared, sectioned and stained with hematoxylin and eosin (H&E) as previously described [17, 18]. For each artery the cells in 10 random images of one tissue section captured at an original magnification of 400x were counted as either single or multinucleated by one blinded observer using ImageJ. For the staining of US11 positive cells, tissue sections were incubated with a mouse antibody to HSV-1 US11 and visualized using a goat anti-mouse horse radish peroxidase diaminobenzidine detection kit (DAKO). The sections were counter stained with hematoxylin. Images were captured with an Olympus DP72 digital camera attached to an Olympus BX51 microscope (Olympus, Center Valley, PA) at original magnifications of 400 and 1000x.

### Assay for cellular release of R7020

SMCs were seeded on glass slides and cultured as described above in “R7020 infection and imaging of SMCs”. On the third day the SMCs were infected with R7020 at 0.1, 1 and 10 MOI for 10 min in DMEM supplemented medium and then washed three times with PBS. For each set of experiments two slides were infected for each MOI. In addition a mock infection was performed with no virus. Cells were then cultured in supplemented DMEM medium. Three sets of experiments were performed. A 72 h time course was performed with the media titrated every 24 h post infection (p.i.). Plus two 24 h experiments were performed. One in which the media were titrated at 12 and 24 h p.i., and another in which the media were titrated at 18 and 24 h p.i.. For each MOI, in each one of these three experiments, one of the two slide’s culture medium was titrated at each time point with a complete medium change each time. The other slide was left undisturbed until the end time point at which time the medium was titrated. Immediately after collection, the medium was serially diluted in 199v and 1 ml of each dilution was added to a T-25 culture of confluent Vero cells. The cells were incubated for 2 h with gentle shaking every 30 min. After which the media were changed to 199ö and the cells were cultured for 3 days. Plaques were counted after the cells were fixed with methanol and stained with giemsa stain.

### Viability assay

SMCs were seeded at a density of 1.25 x 10^4^ cell/cm^2^ in 96 well plates coated with rat tail collagen (BD, Cat. No. 3554236, Medford, MA; 10 μg/cm^2^). They were cultured and cell density was approximated for MOI calculations as described in section “R7020 infection and imaging of SMCs”. On the third day the media were aspirated from the 96 well plates and the cells were infected with R7020 at 0, 0.1, 1 and 10 MOI in 100 μl of DMEM supplemented medium with and without TNF-α (10 ng/ml) for 10 min. Three wells were used for each condition. The cells were then washed twice with PBS and cultured for 24 and 72 h in DMEM supplemented medium with and without TNF-α (10 ng/ml), matching infection conditions. After which, the cells were labeled using the live/dead viability/cytotoxicity kit (Life Technologies, Cat. No. L-3224, Grand Island, NY) as recommended by the supplier. Calcein AM and ethidium homodimer-1 were used at concentrations of 1 and 2 μM, respectively. Fluorescent intensity was measured at 485/530 nm (Abs/Em) for calcein AM and at 530/645 nm (Abs/Em) for ethidium homodimer-1.

### Isolation and labeling of PBMCs

The University of Chicago Biological Science Division Institutional Review Board approved all aspects of the experiments in this paper involving PBMCs including their isolation from healthy donors (Protocol number 12–0179). Blood (40 ml) was drawn after obtaining written consent from healthy subjects following the approved protocol. PBMCs were isolated with Histoplaque-1077 (Sigma-Aldrich, Cat. No. 10771, St. Louis, MO) as recommended by the vender. Cell yield was quantified using a hemocytometer prior to being labeled with calcein AM (BD, Cat. No. 354216, Bedford, MA; 10 μM) at 37°C for 30 min in RPMI (5x10^6^ cell/ml). The PBMCs were then washed twice with 10 ml of RPMI and counted.

### Imaging of adhered PBMCs

SMCs were seeded at a density of 1.25 x 10^4^ cell/cm^2^ on collagen coated (10 μg/cm^2^) coverslips (Fisher Scientific, Cat. No. 12-548-5m, Pittsburgh, PA). They were cultured and cell density was approximated for MOI calculations as described in section “R7020 infection and imaging of SMCs”. On the third day the SMCs were infected with R7020 at 1 MOI for 10 min. After which they were washed three times with PBS and cultured for 24 and 72 h in supplemented DMEM. After culturing, the medium was removed and the cells were washed three times with PBS. The washed SMCs were incubated with calcein AM labeled PBMCs (4 x 10^6^) in DMEM (400 μl) supplemented with 0.1% FBS for 30 min at 37°C. After the incubation, the cells were washed three times with PBS and fixed in 4% PFA and 0.1% gluteraldehyde for 60 min. Images were captured with a Zeiss Axiovert 100TV inverted epifluorescence microscope (Carl Zeiss Microscopy, Thornwood, NY) with a Retiga EXi CCD camera (QImaging, Surry, BC) run by SlideBook 5.5 software (Intelligent Imaging Innovations, Denver, CO) with a 20x objective.

### PBMC adhesion assays

SMCs were seeded at a density of 1.25 x 10^4^ cell/cm^2^ in 96 well plates coated with rat tail collagen (BD, Cat. No. 3554236 Medford, MA; 10 μg/cm^2^). They were cultured and cell density was approximated for MOI calculations as described in section “R7020 infection and imaging of SMCs”. On the third day the medium was aspirated from the 96 well plates and the cells were infected with R7020 at 0, 0.1, 1 and 10 MOI in 100 μl of DMEM supplemented medium with and without TNF-α (10 ng/ml) for 10 min. Three wells were used for each condition. The cells were then washed twice with PBS and cultured for 24 and 72 h in DMEM supplemented medium with and without TNF-α (10 ng/ml), matching infection conditions. After which the SMCs were washed twice with PBS and incubated with calcein AM labeled PBMCs (1x10^5^ cells/100 μl/well) in DMEM supplemented with 0.1% FBS at 37°C for 30 min. The cells were then washed twice with PBS and the intensity of the fluorescence was measured at 485/528 nm (Abs/Em).

### Statistics

Statistical analyses were performed with SPSS for Windows, Version 8.0 (SPSS Inc, Chicago, IL) with significance at p < 0.05. A randomized block design with a Tukey post hoc test was used for the analysis of the live cells in the viability assays and for PBMC adhesion. A two way ANOVA with a Bonferroni multiple comparisons adjustment for pairwise comparisons was used to analyze the percent of dead cells in the viability assays and the culture media titers in the R7020 replication studies. A one way ANOVA with a Bonferroni post-hoc test was used to analyze the histology cell counts. Data are presented as the average ± standard deviation.

## Results

### Cytopathic effect of R7020 on SMCs

**Fig 1:**
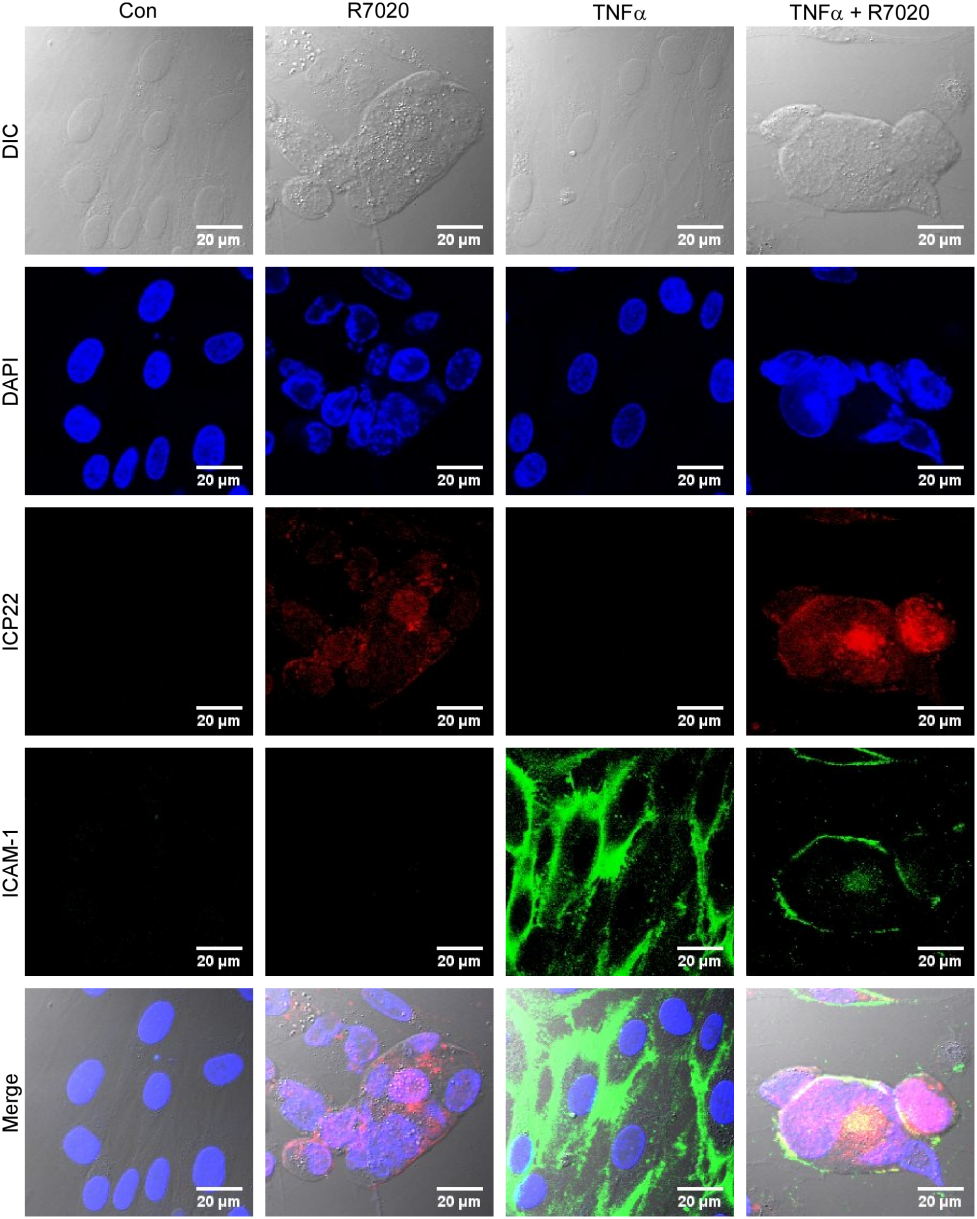
Vascular SMCs infected with R7020 form syncytia *in vitro*. R7020 (1 MOI) infected (R7020) and non-infected (Con) SMCs were cultured for 24 h. Non-infected (TNF-α) and infected cells (TNF-α + R7020) were also cultured in the presence of TNF-α. DIC, DAPI, ICP22 labeled, and ICAM-1 labeled images are shown in rows 1, 2, 3 and 4, respectively with an overlay of all four channels in row 5.

**Fig 2:**
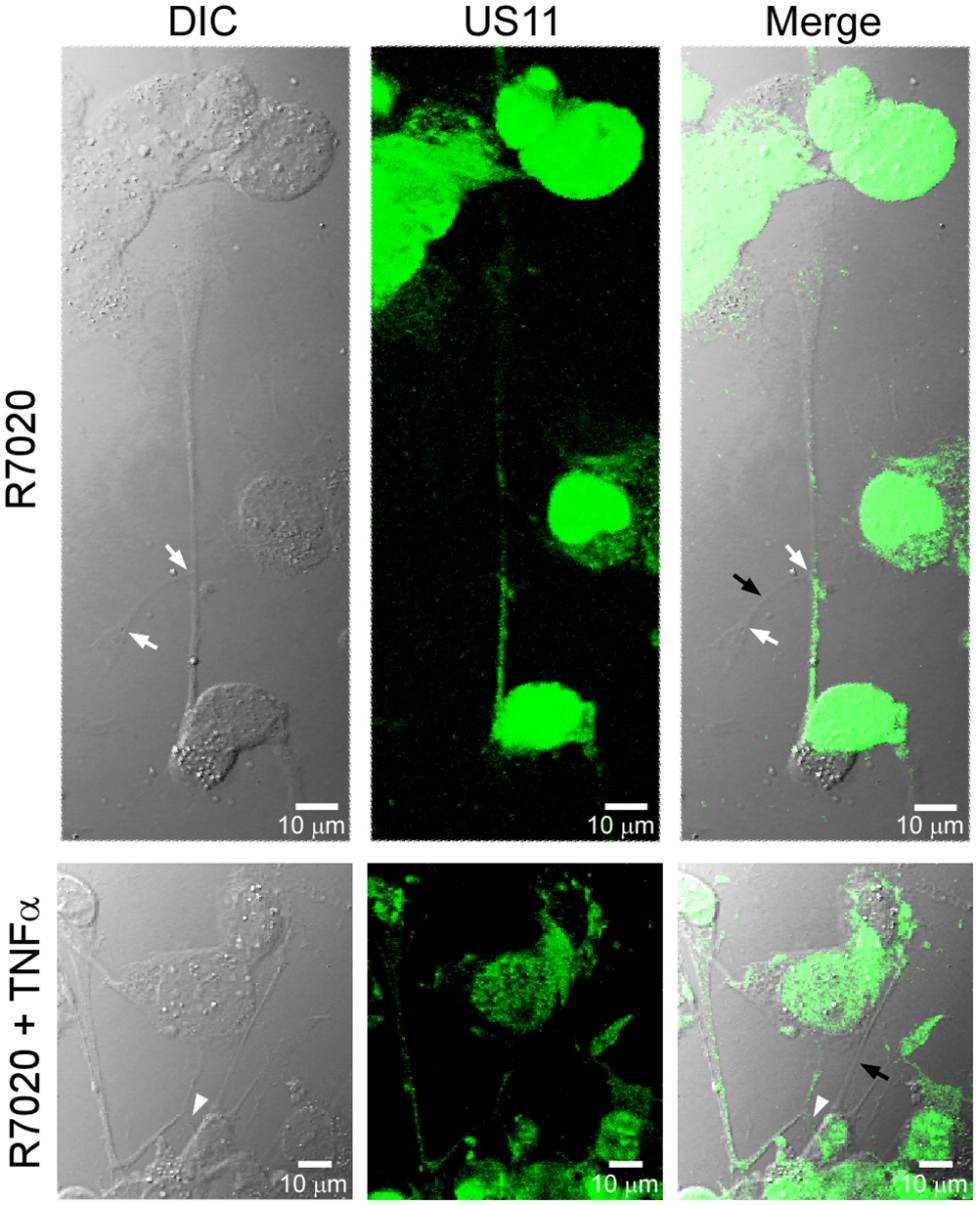
R7020 causes vascular SMCs to form branched protrusions *in vitro*. SMCS infected with R7020 (1 MOI) in the absence (R7020) and presence of TNF-α (R7020 + TNF-α) were cultured 24 h. DIC, US11 labeled and merged images are shown. The white arrows point to branch points in the protrusions. The black arrows point toward protrusions that did not stain positive for US11. The white arrow head points to a protrusion that extends from an infected cell for a short distance and then branches.

To determine the effect of R7020 on SMC morphology cells were infected with R7020 at 1 MOI. Viral infection was confirmed by staining the cells for HSV-1 infected cell protein 22 (ICP22) (Fig 1). ICP22 is a multifunctional protein that localizes to the nuclei of infected cells where it regulates several processes involved in HSV-1 replication including nuclear egress [27]. At 24 h p.i. the cells were undergoing morphological changes. The plasma membranes were contracting inward towards the nuclei the cells causing them to morph from their original elongated spindly shape to ones that were more ovular or circular. Syncytia were also present in the cultures at this point with cell fusion continuing to occur (Fig 1). Multiple filopodia were observed extending from cells and syncytia, some of which branched multiple times and connected with other cells and syncytia (Fig 2). We also examined the effect of R7020 on SMC morphology in the presence of TNF-α as it is produced by SMCs and immune cells at increased levels in diseased vessels [28–32]. To confirm that the cells were stimulated by TNF-α in the presence of R7020 the cells were stained for ICAM-1, a cell surface receptor known to be up regulated by TNF-α [33]. This receptor was expressed on the surfaces of R7020 infected and non-infected SMCs treated with TNF-α but not on the plasma membranes of cells cultured in the absence of TNF-α (Fig 1). TNF-α did not alter the effects of R7020 on SMC morphology for TNF-α stimulated cells also formed syncytia and filopodia (Figs 1 and 2).

**Fig 3:**
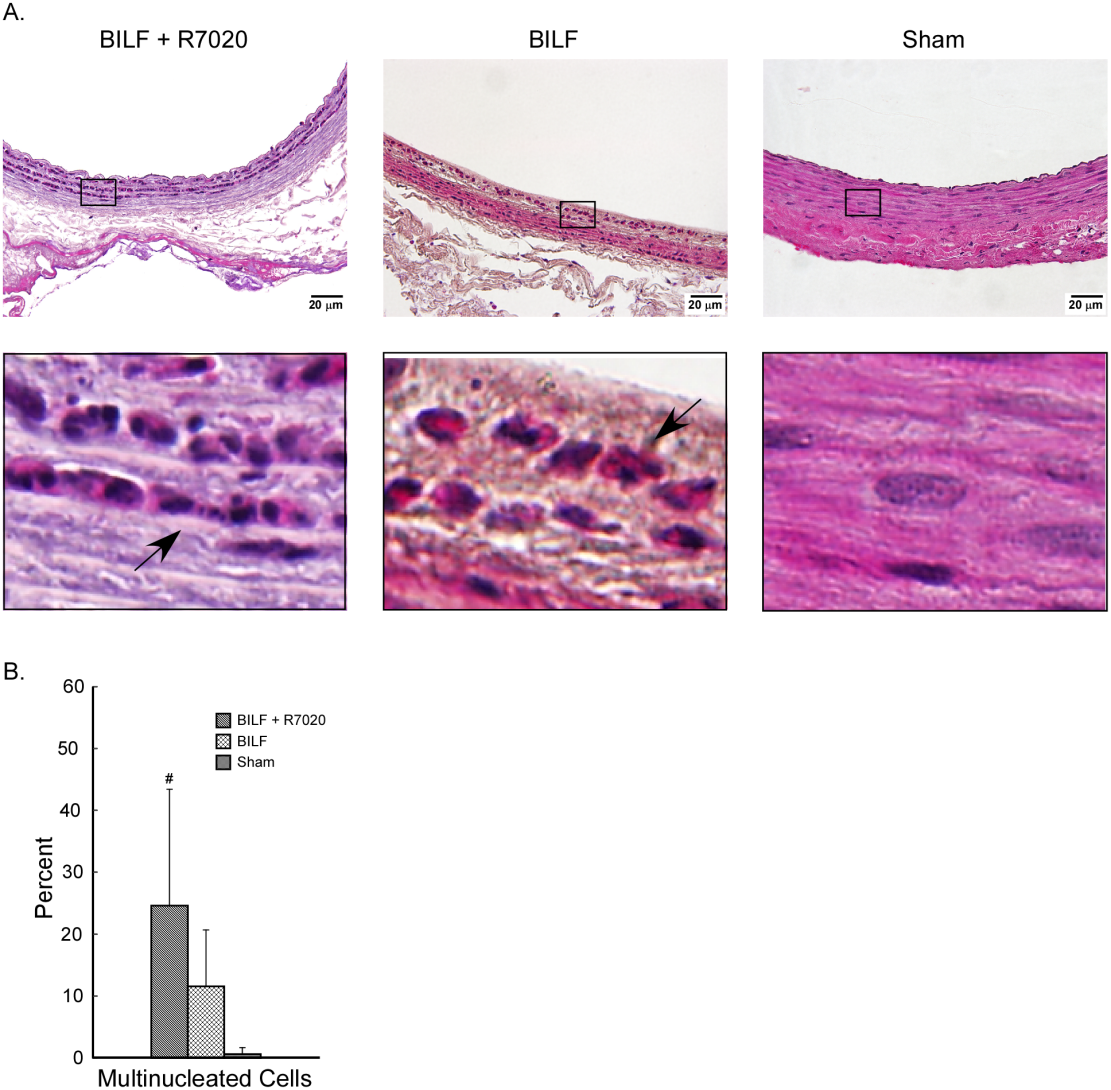
Vascular cells in an *in vivo* balloon injury low flow model 24 h p.i. with R7020. Following injury with a balloon catheter the carotid artery was infected with R7020 (BILF + R7020) or mock infected (BILF) and blood flow was returned at a reduced flow rate for 24 h. The control (Sham) carotid artery was separated from the surrounding tissue but the wound was closed with no arterial intervention and no flow rate alteration. (A) Images of H&E stained carotid artery wall sections with 10X magnified views of the indicated regions. The arrows in the images of BILF + R7020 and BILF tissue sections point to multinucleated cells. (B) Percent of total cells that are multinucleated for each treatment group. A statistically significant difference (p < 0.05) compared to sham is indicated by #.

**Fig 4:**
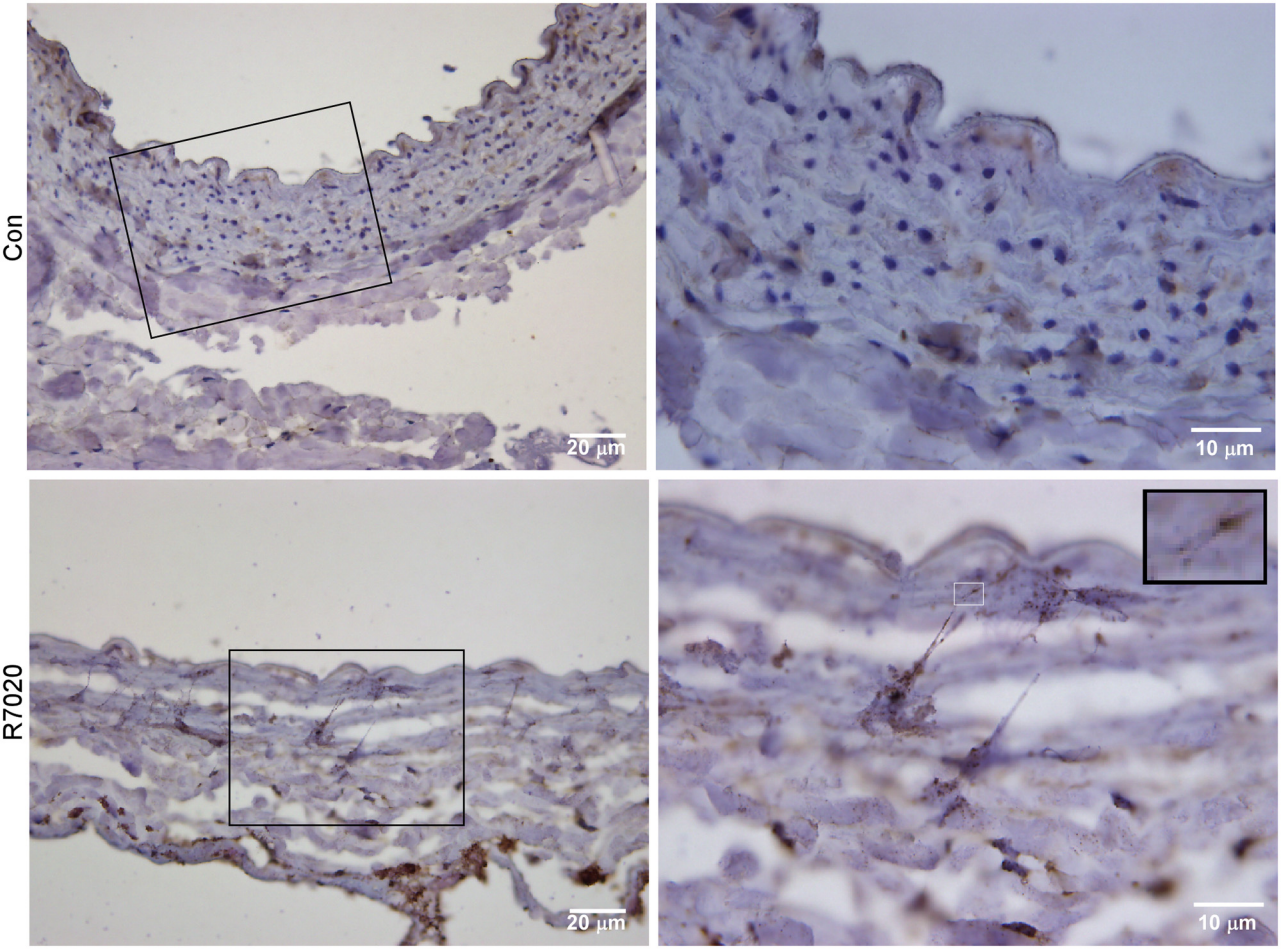
R7020 causes the formation of cell protrusions *in vivo*. Shown are US11 labeled histology sections of a balloon injured R7020 exposed carotid artery exposed for 3 days to low flow (R7020) and a normal carotid artery with no interventions (Con). The images on the left and right were originally imaged at 400 and 1000x magnification, respectively. The branched end of a protrusion can be seen in the enlarged region of the R7020 image (1000x). This insert is a 4x magnified view of the region outlined by the white rectangle.

An *in vivo* balloon injury low flow IH model was used to investigate the effect of R7020 on cells within a pathological arterial wall. After the CCA was injured it was infected with R7020 and blood flow was restored at a reduced rate. Cells in non-infected balloon injured CCAs and in sham treated CCAs were used for comparison purposes. At 24 h multi-nucleated cells were observed in the arteries (Fig 3). In the R7020 infected BILF CCAs 24.6 ± 18.8 percent of the cells were multi-nucleated. This was significantly greater than the percent for the sham treated CCAs (0.6 ± 1.1%). However, there the percent of multi-nucleated cells in the infected and non-infected BILF CCAs was statistically the same (24.6 ± 18.8 vs. 11.6 ± 9.1). The syncytia tended to be larger in the R7020 infected CCAs than in the non-infected CCAs and were located deeper within the vessel walls (Fig 3). After three days, filopodia were observed extending from US11 positive cells in the media of the vessel wall towards the intima (Fig 4). US11 is an HSV-1 protein that localizes to the teguments of viral particles as well as to the nuclei. It was present along the entire length of some of the filopodia and also at the ends of branches that laid on the surfaces of neighboring cells (Fig 4 insert).

### Release kinetics of R7020 in vascular SMCs

**Fig 5:**
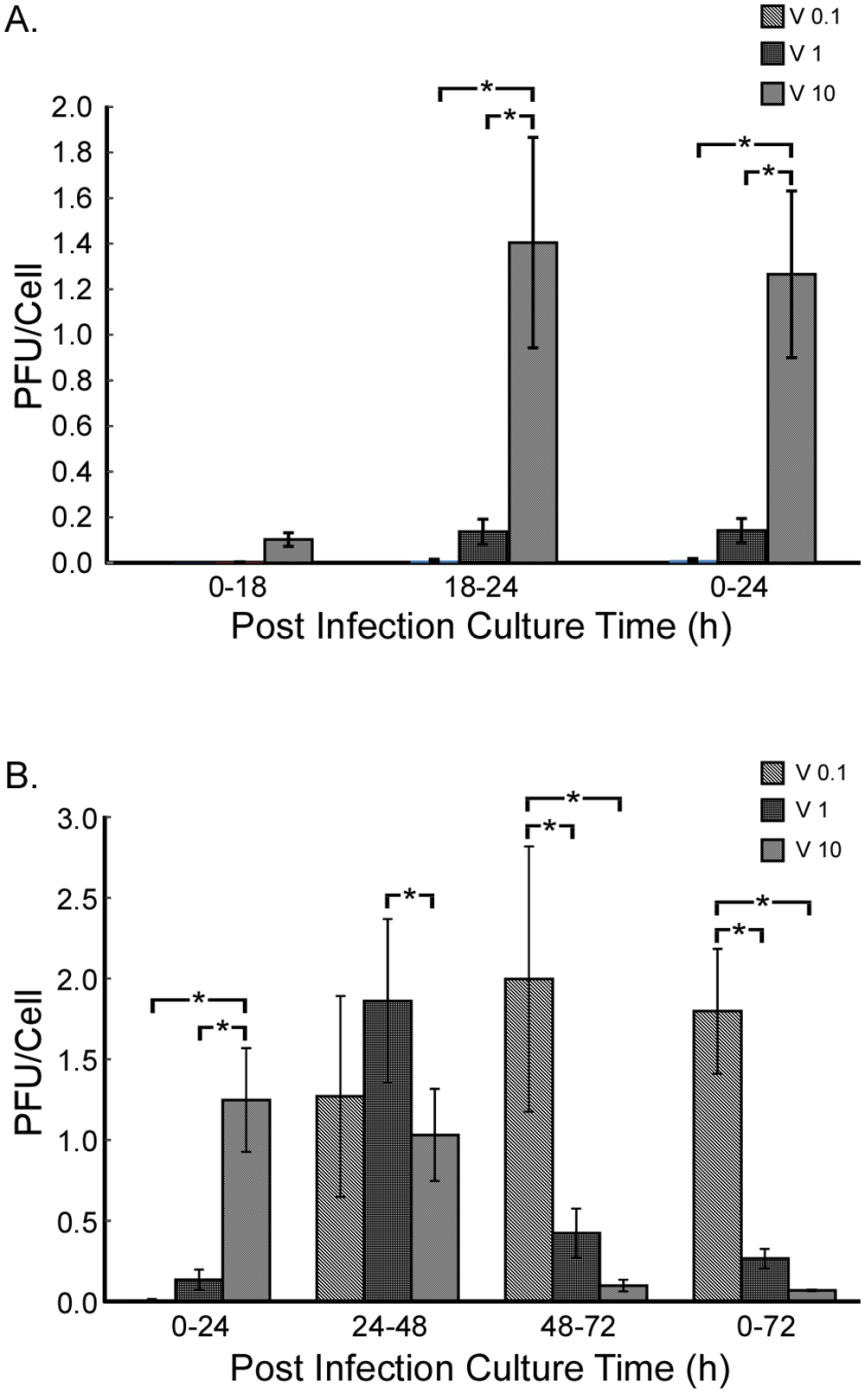
R7020 infected SMC culture media titers. The media used to culture SMCs after infection with R7020 at 0, 0.1, 1 and 10 MOI were titrated using a plaque assay. The number of cells present during the initial infection was used to normalize the titers. (A) A short time course to determine when the virus begins to be released by the cells into the culture medium. (B) R7020 titer levels in a 72 h time course study. A statistically significant difference (p < 0.05) in titer levels is indicated by *.

To determine the initial release kinetics of R7020 from SMCs cells were infected with 0, 0.1, 1 and 10 MOI of R7020 and the culture media were titrated at 12, 18 and 24 h p.i.. At 12 h p.i. R7020 could only be measured in the culture media of cells infected at 10 MOI (data not shown). By 18 h p.i. the virus was also present in the media of the 1 MOI infected cultures (Fig 5A). For the cultures infected at 0.1 MOI R7020 was not detectable until 24 h p.i.. Thus the initial release kinetics of the virus from the SMCs was dependent upon viral dose. The amount of virus released during the first 24 h was also dose dependent. The 1 and 10 MOI titers of the 18–24 h p.i. cultures were 12 and 126 times greater than the 0.1 MOI titer. Similarly, the 0–24 h p.i. culture titers for the 1 and 10 MOI infections were 11 and 99 times greater than the 0.1 MOI culture titer. The virus was not detected in the culture medium of the non-infected SMCs.

We also investigated the long-term release kinetics of R7020 with these MOIs titrating the culture media at 24, 48 and 72 h p.i.. Similar to the initial viral release, the long term release profile was a function of time and dose (Fig 5B). At 0.1, 1 and 10 MOI the viral titers were highest at 72, 48 and 24 h p.i., respectively. At 24 h p.i., the 10 MOI infection resulted in a titer level that was at least nine times greater than those of the 0.1 and 1 MOI infections. These low infection levels resulted in low virus titers during the first 24 h. At 48 h p.i. cells infected at 10 MOI continued to release the virus at an elevated level with no significant change from 24 h p.i.. However, the culture medium titers of the cells infected at 0.1 and 1 MOI had increased. And the titer for the 1 MOI infection was now significantly greater than that for the 10 MOI infection. The 1 MOI infection only resulted in an elevated virus titer at one time point, 48 h p.i., as the level declined at 72 h p.i.. The titer of the 10 MOI infection also declined sharply at this time point. In contrast, the titer of the 0.1 MOI infected cells continued to increase at 72 h p.i., although not significantly, and was 4.7 and 20 times greater than the 1 and 10 MOI titers, respectively. Thus, the highest and lowest infection levels, 10 and 0.1 MOI, resulted in culture medium titers that were sustained for longer time periods than that of the intermediate infection level, 1 MOI. The highest infection level also resulted in the quickest release of large amounts of virus, whereas the lowest infection level resulted in the slowest. The removal of the media for analysis had no effect on later titer levels as the titers at 72 h p.i. were the same with or without medium changes.

Although these are *in vitro* results, they suggest that R7020’s *in vivo* release kinetics will depend on the dose used to infect the vessel wall. Since the formation of IH is an orderly process that follows a sequence of events, the time at which R7020 reaches its peak level and the amount of time it takes for the virus to run its course will undoubtedly impact the effectiveness of the virus on inhibiting IH. Thus viral dose stands to be an important parameter in the optimization of R7020’s inhibitory effect.

### Effect of R7020 on SMC viability

**Fig 6:**
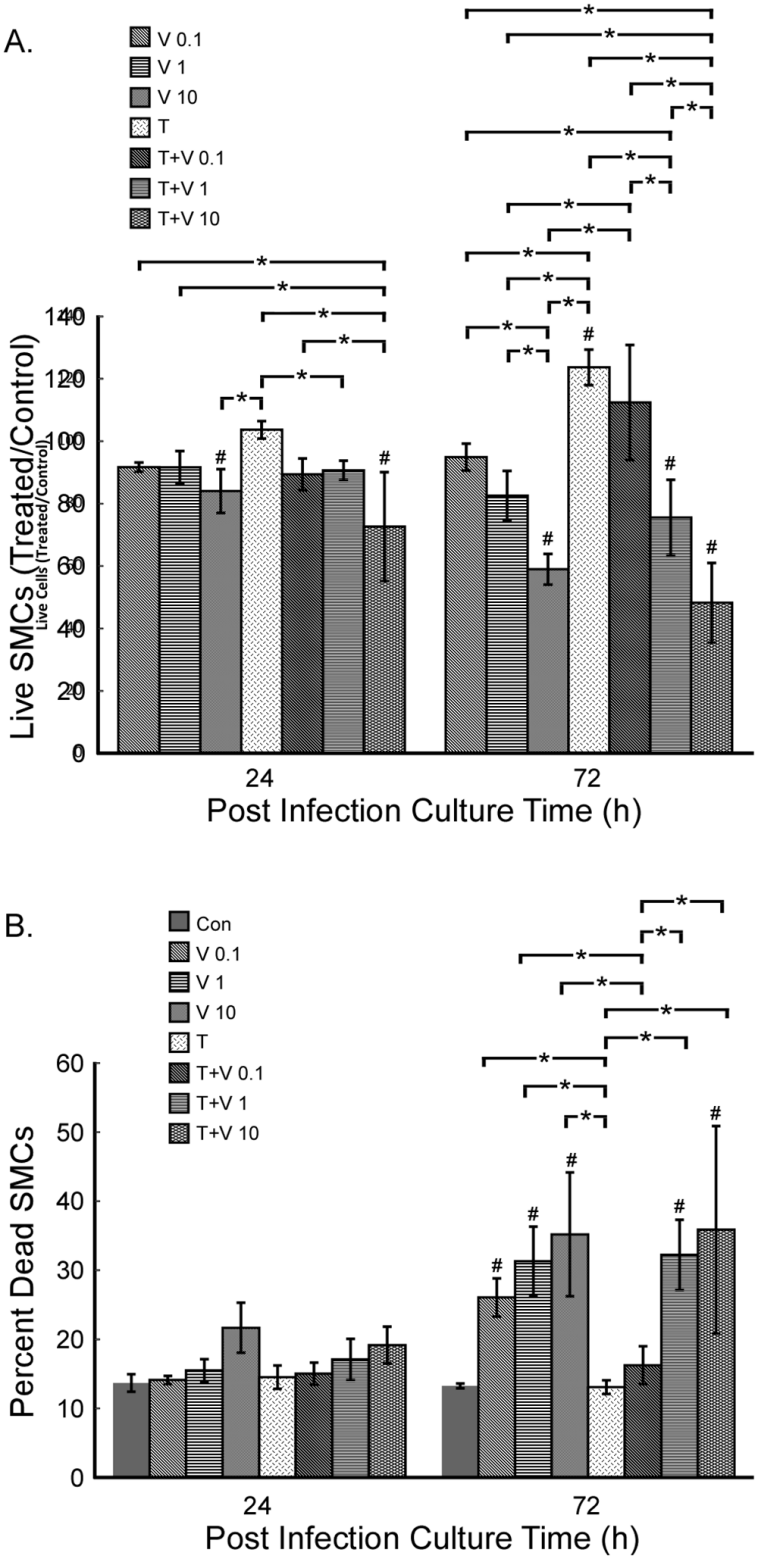
Live and dead SMC levels post infection with R7020. Vascular SMCs were infected with R7020 at 0, 0.1, 1 and 10 MOI in the presence and absence of TNF-α. The cultures were assayed for live and dead cells 24 and 72 h p.i.. (A) The number of live cells were normalized by the number of live cells in the control non-infected SMC culture. (B) The number of dead cells is presented as a percent of the total number of cells, live plus dead, in the respective culture. Abbreviations used: T = TNF-α, V = virus, Con = control non-infected cells cultured in the absence of TNF-α. Statistically significant differences (p< 0.05) for live and dead cells are indicated by *. A # indicates a statistically significant difference compared to control.

To determine the effects of R7020 on cell viability live-dead assays were performed 24 and 72 h p.i. on SMC cultures infected at 0, 0.1, 1 and 10 MOI. In the absence of TNF-α the number of live cells was only affected by the highest R7020 infection level, 10 MOI, decreasing it by 16 and 42% relative to control at 24 and 72 h p.i., respectively (Fig 6A). In comparison, in TNF-α treated cells R7020 at 1 and 10 MOI caused significant decreases. Cell levels decreased 12 and 29% for 1 and 10 MOI, respectively, at 24 h p.i. and 34 and 61%, respectively at 72 h p.i. compared to the TNF-α control. TNF-α caused a significant 27% increase in the number of live cells in the non-infected cultures at 72 h p.i. but had no effect on infected cells. Due to TNF-α’s ability to increase SMC proliferation, R7020 had the greatest effect on SMC number in TNF-α treated cultures where at the higher infection doses it negated the effect of TNF-α.

At 24 h p.i. there were no significant differences in the percentage of dead cells for the different treatment conditions (Fig 6B). However, from 24 to 72 h p.i. the percentage of dead cells significantly increased in the R7020 infected cultures but not in control. The increases were 82, 103 and 68% for 0.1, 1 and 10 MOI, respectively. In addition, at 72 h p.i. there was a greater percent of dead cells in the R7020 infected cultures than in control at all three doses. In TNF-α treated cells only the two highest infection levels increased the number of dead cells at 72 h. The percentage increased 84 and 79%, from 24 to 72 h p.i. for 1 and 10 MOI, respectively relative to TNF-α alone. TNF-α did not have a significant effect on the percent of dead cells in R7020 infected cultures.

The results of these live dead assays indicate that the observed decreases in virus titers with time for the 1 and 10 MOI R7020 doses was not due solely to a decrease in the number of live cells or an increase in the number of dead cells. For although there are fewer live cells and more dead cells at 72 h for these infection levels, the differences between them and those for the 0.1 MOI dose are too low to account for the differences in the culture media titers.

### Adhesion of PBMC to R7020 infected SMCs

**Fig 7:**
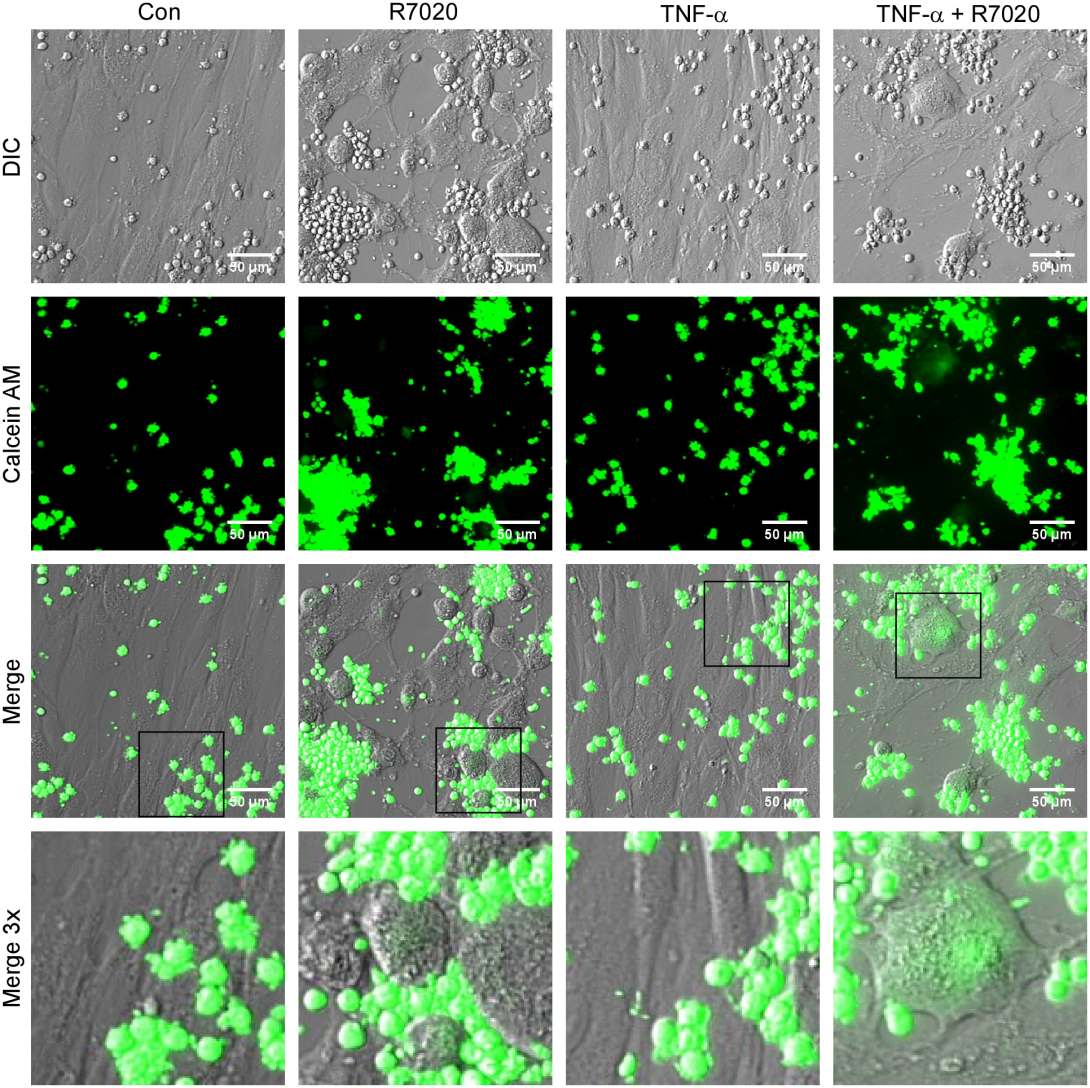
Adhesion of PBMCs to SMCs infected with HSV-1 R7020. Vascular SMCs, 72 h p.i. with R7020 (1 MOI) in the presence and absence of TNF-α, were incubated with calcein AM labeled PBMCs. DIC and fluorescent images are shown in rows one and two, respectively with overlays of the two channels in row three. In the fourth row are magnified (3X) images of the rectangular region outlined in the above overlay. Note the fluorescence of the SMCs that were infected with R7020. Abbreviations used: Con = non-infected SMCs, R7020 = SMCs infected with R7020, TNF-α = non-infected SMCs cultured in the presence of TNF-α, TNF-α + R7020 = R7020 infected SMCs cultured in the presence of TNF-α.

To investigate if R7020 alters interactions between immune cells and SMCs the effect of R7020 on PBMC adhesion was studied by incubating fluorescently labeled PBMCs with infected cells. At 24 and 72 h p.i. PBMCs had attached to non-infected and infected cells as well as syncytia in the absence and presence of TNF-α (S1 Fig and Fig 7). However the PBMCs in the infected cultures tended to form larger cell clumps. In addition, fluorescent SMCs and syncytia were observed in R7020 infected cultures but not in the non-infected cultures suggesting that the PBMCs had fused with the infected cells.

**Fig 8:**
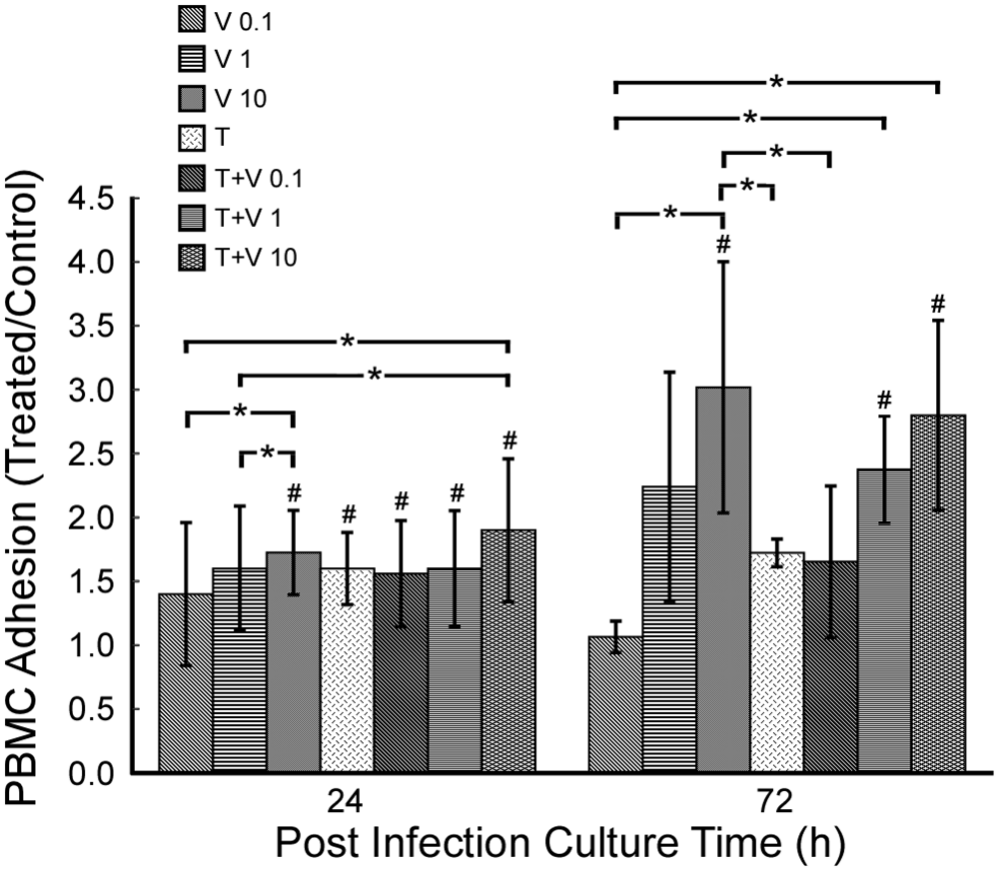
Adhesion of PBMCs to R7020 infected SMCs 24 and 72 h p.i.. Fluorescently labeled PBMCs were incubated for 30 min with R7020 (0, 0.1, 1 and 10 MOI) infected SMCs 24 and 72 h p.i. in the presence and absence of TNF-α. Fluorescent intensities normalized by non-infected cells cultured in the absence of TNF-α are plotted in the graph. Abbreviations used: T = TNF-α, V = virus. Statistically significant differences (p< 0.05) in normalized fluorescent intensities are indicated by *. A # indicates a statistically significant difference compared to control.

We quantified the adhesion of PBMCs to infected SMCs for infection levels of 0, 0.1, 1 and 10 MOI at 24 and 72 h p.i. (Fig 8). In the absence of TNF-α only R7020 at an infection of 10 MOI caused a significant increase in the adhesion of PBMCs compared to control at 24 and 72 h p.i.. However, there was a dose effect at 72 h p.i.. When TNF-α stimulated cells were infected, R7020 did not significantly alter adhesion at either time point although adhesion levels did increase with dose at 72 h p.i.. Although their effect was equivalent at 24 h p.i., at 72 h p.i. R7020 at 10 MOI in the absence of TNF-α had a greater effect on PBMC adhesion than TNF-α with 75% more PBMCs adhering.

## Discussion

This study suggests that the HSV-1 strain R7020 uses several mechanisms to avoid the immune system and inhibit IH in the vasculature. Our results indicate that R7020 evades immune cells by primarily disseminating via cell-to-cell mechanisms instead of by diffusion through the aqueous extracellular environment. Consequently, these same mechanisms may contribute to the ability of R7020 to inhibit IH. For example, R7020’s induced fusion of SMCs for viral spreading may also be a key mechanism by which R7020 inhibits SMC proliferation in injured blood vessels. Similarly, R7020 may limit the effect of the immune system on IH by inducing the fusion of immune cells with infected cells and syncytia.

Viruses have developed several methods to infect cells that involve direct contact between infected and non-infected cells allowing the viruses to remain cell associated after egress until they infect new cells [34, 35]. Cell to cell spread has many advantages over dissemination by diffusion through extracellular fluid including rapid viral spread and evasion of immune defenses [34, 35]. HSV-1 is known to largely remain attached to cells after egression. It is released by a form of exocytosis and disseminates efficiently via cell to cell spread. Using cell polarity to direct viral egress the virus is able to take advantage of epithelial cell-cell junctions and neuronal synapses to spread rapidly and under the radar of the immune system. Many viruses including HSV-1 also induce the formation of intercellular filopodial bridges or cytonemes, and nanotubes to connect infected and non-infected cells. Forms of heparan sulfate (HS) modification play a critical role in viral entry, replication and trafficking in other viral infections including cytomegalovirus and hepatitis B virus [36, 37]. The modification of HS to 3-*O*-sulfated heparan sulfate (3-*O*S HS) by 3-*O*-sulfotransferases (3-*O*STs) plays an important role in the induction of filopodia formation by HSV-1 [38], as the expression of 3-*O*ST increases the number of filopodia on cells expressing HS [39–41]. Viruses will travel either on the surface or within the protrusions as they spread from cell to cell [34, 42–46]. HSV-1 induces the formation of filopodia in several different cell types [43–46]. In this study the *in vitro* and *in vivo* cytopathic effect of R7020 on SMCs included the formation of filopodia. Many of the protrusions extended from one cell to another suggesting that viral dissemination occurs via cell to cell spread as herpes viruses are known to travel along protrusions. During cell infection the virus moves towards endocytotic hot spots at the cell body. Whereas, during viral egress HSV-1 uses protrusions extending from infected cells to move away from the cell bodies where they replicated and towards the target cells they will infect [43]. La Boissiere et al. reported viruses traveling down the projections to bulbous ends that are anchored in the membranes of uninfected adjacent cells [47]. Leeuween et al. localized nonmuscle myosin II (NMIIA), a non-processive motor that may play a role in virus egress trafficking, to HSV-1 induced protrusions [48]. Associated with the NMIIA were particles containing VP22, a major HSV-1 tegument protein suggesting that viruses are being transported down the protrusions [48]. Similar to Boisiere’s observation we also observed projections which split into bulbous ends that overlaid cells and stained positive for a HSV-1 tegument protein *in vivo*. And similar to Leeuween et al. we observed *in vivo* positive staining for HSV-1 tegument protein US11 along the length of R7020 induced protrusions [48]. Collectively these studies suggest that R7020 induces SMCs in vascular vessels to form protrusions that extend between cells to use as conduits for travel between infected and noninfected cells. Indicating that in blood vessels the virus uses SMC protrusions to spread efficiently and avoid the immune system.

Viruses also spread cell to cell by inducing the fusion of infected and noninfected cells to form syncytia. Only viable cells are incorporated into syncytia and after they are incorporated they can no longer replicate [49]. Sylwester *et al*. have shown using electron microscopy that syncytia formed during HIV infection are very well organized and only grow as additional cells fuse [50]. Wild type HSV-1 expresses the syncytial phenotype *in vivo* but rarely *in vitro*. However, several HSV-1 strains do induce the formation of syncytia *in vitro*. In order for the strains to be able to induce the formation of syncytia they must express HSV-1 envelope glycoproteins gB, gD, and the heterodimer gH/gL [51–54]. In addition a mutation must occur in one of four HSV-1 genes, gB, gK, UL20, or UL24 [51, 55–69]. The strain used in these studies, R7020, lacks the UL24 gene confirming the syncytial phenotype on it. Cell fusion is dependent upon cell type and requires a receptor for HSV-1 gD on the plasma cell membrane. Inhibitor studies have shown that phosphoinositide 3 kinase (PI3K) activity is also required for optimal cell fusion and interestingly also for the induction of filopodia formation by HSV-1 and other members of the herpesvirus family [70]. However, the mechanism by which cell fusion occurs has yet to be completely delineated [71, 72]. In this study each of the viral infection levels used, 0.1, 1 and 10 MOI, caused SMCs to form syncytia *in vitro*. In addition, multinucleated cells were also present in R7020 infected vascular vessels *in vivo*. Our morphological data along with our virus release data, where culture media changes had no effect on titers at later time points, suggest that R7020 primarily infects SMCs via cell to cell spread which is efficient and avoids the immune system.

We have previously shown that R7020 inhibits IH in a balloon injury low flow model [19]. The results from this study indicate some of the mechanisms R7020 may use to accomplish this including ones that are a consequence of R7020 spreading. Perhaps the most significant way R7020 limits IH is by its infection of SMCs and their subsequent formation of syncytia. For once the cells fuse together they will no longer be able to proliferate [49] and will ultimately die. This technique is similar to that of HIV which uses the induction of syncytia formation as a major mechanism to kill T-cells [50]. A second mechanism may involve the targeted infection of SMCs in the intima via protrusions. R7020 induced the formation of protrusions by SMCs both *in vitro* and *in vivo*. *In vivo*, the protrusions were concentrated in the media and intima of the blood vessel wall. They were primarily oriented perpendicular to the luminal surface of the blood vessel, which is orthogonal to the direction SMCs orient in a healthy blood vessel. Thus they tended to extend from the media towards the lumen. This is suggestive of a scenario where a percentage of the media SMCs are infected during the initial exposure of the blood vessel to R7020. While the virus is replicating uninfected cells proliferate and migrate into the intima in response to the injury. As the virus is released it travels down the protrusions targeting the SMCs in the intima that would otherwise continue to proliferate and synthesize extracellular matrix leading to IH if left unchecked.

The immune system is another avenue through which R7020 may inhibit IH. T-cells and macrophages play significant roles in the regulation of SMCs and in the development of IH. Lymphocytes adhere to the wall of denuded arteries within 1 h with both CD4+ and CD8+ T-cells adhering. In endothelial denudation models lymphocytes have a protective role decreasing the number of proliferating SMCs in the injured artery and decreasing intimal thickening [73–75]. Interferon-γ (IFN-γ), which is secreted by activated T-cells, inhibits SMC proliferation *in vitro* and in *in vivo* lesions where injury induced SMC proliferation is occurring in the intima [73, 76]. However when IFN-γ is released late it further exacerbates intimal thickening [77]. The likely source of IFN-γ at the later time points is the macrophage. In contrast to lymphocytes monocytes/macrophages promote intimal thickness after denudation [75, 78–82]. In our study the adhesion of PBMCs to R7020 infected SMCs increased with time. In addition, there was transfer of fluorescent label from PBMCs to syncytia suggesting that the PBMCs had fused with them. Together this suggests that macrophages, which arrive later than T-cells to the injured vessel, would be more likely to have their levels decreased via fusion with R7020 infected SMCs than the T-cells. If this occurs the reduction in the number of active macrophages could potentially decrease the severity of the IH [83, 84].

TNF-α, an inflammatory cytokine increases the severity of IH. It is expressed at elevated levels by SMCs and immune cells in balloon injured arteries, diseased vein grafts and atherosclerotic lesions [28–32, 85]. TNF-α expression by medial SMCs proceeded their proliferation in a rabbit balloon injury model [86]. Mice lacking TNF-α develop 14 fold less IH area than normal mice in a low flow CCA ligation IH model, while the over expression of TNF-α causes an increase in IH [31]. Wang et al. showed that TNF-α stimulates spindle shaped SMCs to proliferate but causes epithelioid shaped cells to undergo apoptosis [87]. In agreement with this and what others have reported [88, 89], we observed a significant increase in SMC proliferation with TNF-α treatment. However, this increase was inhibited by R7020 in a dose dependent manner. This may in part be a result of the IFN self-defense mechanism of cells in response to viruses. IFN-β has been shown to inhibit TNF-α, PDGF and IL-1 induced SMC proliferation [90]. The synthesis of IFN-β is increased by viruses through pathways [91] that activate IFN regulatory protein 3 (IRF3) which binds to the IFN-β promoter producing the first wave of IFN [92]. The paracrine/autocrine binding of secreted IFN with its receptor leads to a second larger wave of IFN synthesis [93]. The secreted IFN-β then acts as either an autocrine factor or a paracrine factor on noninfected SMCs and inhibits their proliferation.

In this study TNF-α did not alter the effects of R7020 on PBMC adhesion. TNF-α mediates monocyte adhesion to SMCs by increasing ICAM-1 expression [33]. In agreement with this we observed an increase in the adhesion of PBMCs to TNF-α stimulated SMCs. R7020 at 10 MOI also caused a similar increased in PBMC adhesion at 24 h. SMCs infected with R7020 did not express ICAM-1, but when the infected cells were treated with TNF-α ICAM-1 was present on the plasma membrane. The lack of ICAM-1 on the surface of R7020 infected cells in the absence of TNF-α indicates that R7020 increases PBMC adhesion through a different pathway than TNF-α. However, paradoxically R7020 and TNF-α did not have an additive effect on PBMC adhesion.

Overall this study provides evidence that the HSV-1 strain R7020 may be using multiple mechanisms to inhibit IH as we hypothesized. Further studies are necessary to determine the importance of each one and identify the key factors. The results of this study also indicate that the viral dose used to prevent IH in vein grafts and other vessels may be critical for maximum effect. It needs to be carefully studied in clinical trials as dose may determine not only peak viral level but more importantly it may determine when it occurs. Timing is a critical factor when treating diseases like IH which follow an orderly sequence of events some of which may be more susceptible to R7020 than others. Previously we have shown that R7020 inhibits IH *in vivo*, here we have identified several mechanisms by which this may occur. Further investigation is needed to delineate the pathways and identify the key factors and parameters.

## Supporting Information

S1 Fig Adhesion of PBMCs to SMCs 24 hours post infection with HSV-1 R7020. Vascular SMCs were incubated with calcein-AM labeled PBMCs 24 h post infection with R7020 (1 MOI) in the presence and absence of TNF-α. DIC and fluorescent calcein-AM images are shown in rows one and two, respectively with overlays of the two channels in row three. In the fourth row are images of the region indicated in the above overlay magnified 3X. Note the fluorescence of the SMCs that were infected with R7020. Abbreviations used: Con = non-infected SMCs, R7020 = SMCs infected with R7020, TNF-α = non-infected SMCs cultured in the presence of TNF-α, TNF-α + R7020 = R7020 infected SMCs cultured in the presence of TNF-α.
(TIF)
