# Supplementary figures and images for: Evidence for the Use of Multiple Mechanisms by Herpes Simplex Virus-1 R7020 to Inhibit Intimal Hyperplasia
Source: PLoS One. 2015 Jul 1;10(7):e0130264. doi: 10.1371/journal.pone.0130264 (PMC4488439; doi:10.1371/journal.pone.0130264)

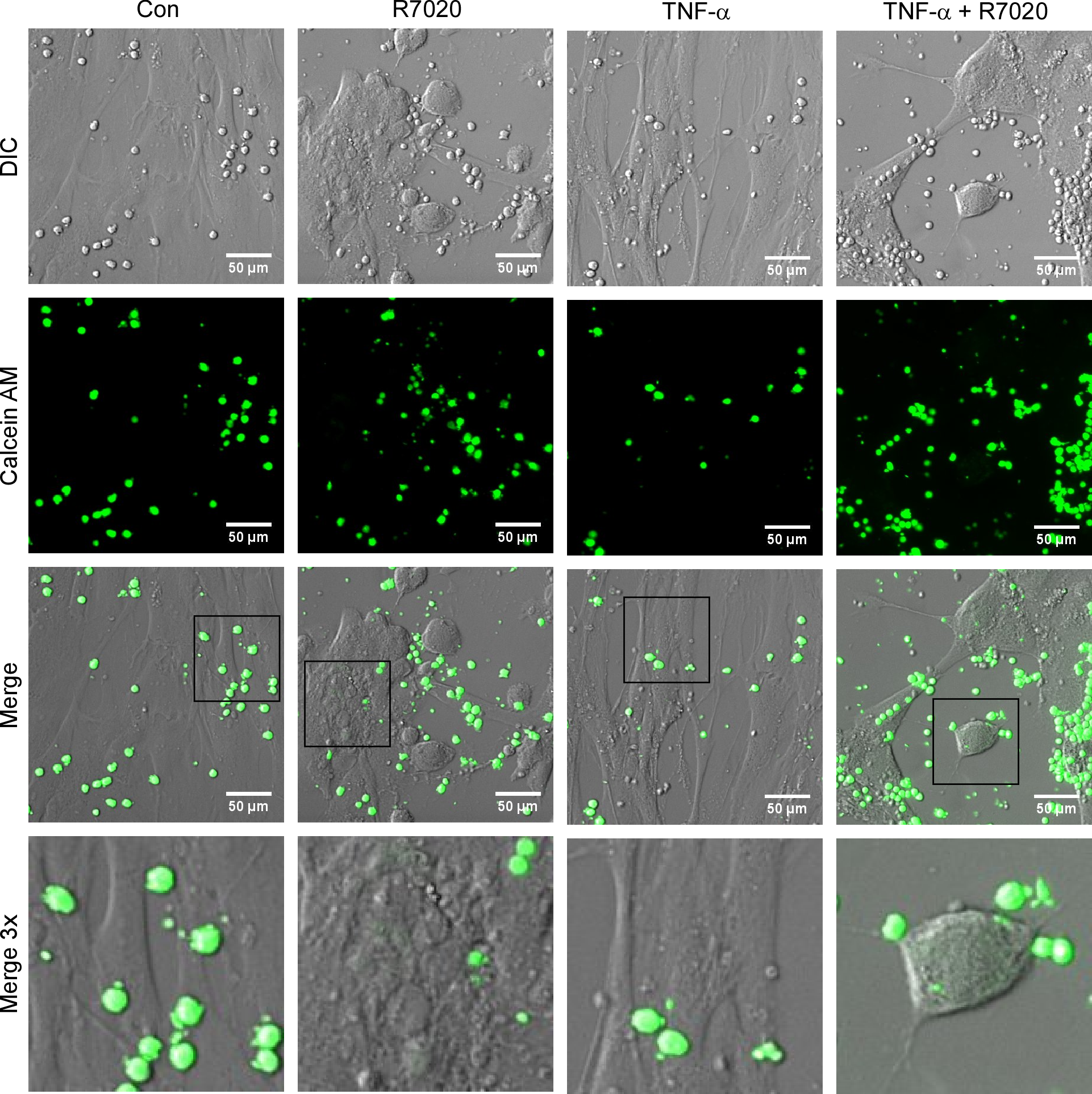

Supplement: S1 Fig — Vascular SMCs were incubated with calcein-AM labeled PBMCs 24 h post infection with R7020 (1 MOI) in the presence and absence of TNF-α. DIC and fluorescent calcein-AM images are shown in rows one and two, respectively with overlays of the two channels in row three. In the fourth row are images of the region indicated in the above overlay magnified 3X. Note the fluorescence of the SMCs that were infected with R7020. Abbreviations used: Con = non-infected SMCs, R7020 = SMCs infected with R7020, TNF-α = non-infected SMCs cultured in the presence of TNF-α, TNF-α + R7020 = R7020 infected SMCs cultured in the presence of TNF-α. (TIF) [file pone.0130264.s001.tif]
